# Professional nurses’ perspectives of an ideal performance management process

**DOI:** 10.4102/hsag.v29i0.2595

**Published:** 2024-10-15

**Authors:** Sibonelo Ndlovu, Neltjie C. van Wyk, Ronell Leech

**Affiliations:** 1 Department of Nursing Science, Faculty of Health Sciences, University of Pretoria, Pretoria, South Africa

**Keywords:** appreciative inquiry, ideal, nurse manager, performance management, professional nurse

## Abstract

**Background:**

A well-managed performance management process can significantly influence professional nurses’ job satisfaction and improve patient outcomes. Conversely, ineffective management of the process can lead to demotivation of the nursing staff.

**Aim:**

This study aimed to understand professional nurses’ perspectives on an ideal performance management process.

**Setting:**

The study was conducted in a designated hospital in South Africa owned by a private healthcare group. The study population consisted of professional nurses involved in a performance management cycle.

**Methods:**

A descriptive qualitative research design with an appreciative inquiry approach was employed. Participants were selected through purposive sampling. Five focus groups of five professional nurses each were used to conduct interviews. The interviews lasted between 60 min and 80 min. The number of interviews conducted provided sufficient data for data saturation. The data were analysed using thematic analysis and the 5-D model of appreciative inquiry.

**Results:**

Trusting relationships between management and professional nurses play an essential role in ensuring a tailored performance management process. Training of both professional nurses and managers, a sound reward system for high-performing employees, and a fair and transparent process that addresses challenges and promotes opportunities can promote a positive work environment.

**Conclusion:**

Professional nurses require support from their managers and top management. Involving professional nurses in the planning and implementation can ensure proper relationships exist and that constraints are appropriately addressed.

**Contribution:**

Implementing the findings can improve the competencies of both managers and professional nurses to maintain a positive performance management process.

## Introduction

Performance management is a systematic process for establishing standards, ensuring improvements, evaluating outcomes and recognising achievement. It is usually conducted to manage and improve the performance of the staff and enhance the overall hospital efficiency (Ahmed, Fakhry & Kamel 2024). Setting goals across the board, sharing them with staff, encouraging goal-setting among them and using feedback to assess progress are all expected of organisations (Nwokeocha 2024). Positive work attitudes among employees can result from effective performance appraisal that combines justice and fairness successfully (Brefo-Manuh & Anlesinya 2023).

The current performance management system suggests that healthy work environments and good relationships with management may increase nurses’ satisfaction with their performance management process (Madlabana & Petersen 2020). According to Sopiah et al. (2020), fair performance management influences work commitment and subordinates’ performance to the extent that subordinates will generally over-perform for the affirmation of their managers. Professional nurses who are satisfied with the institution’s performance management process tend to stay in the organisation and not seek other opportunities elsewhere (Parveen et al. 2020; Rehman, Din & Kashif 2022). The fair execution of performance management corresponds with nurses’ commitment to the organisation (Amarneh & Muthuveloo 2020; Hamdeen, Elewa & Mohamed 2022). Ayi and Korang (2024) maintained that aligning a sound reward system with organisational goals may enhance productivity and foster a motivated and engaged workforce.

Managers’ transparency with the techniques for assessment of performance and the distribution of incentives may also enable professional nurses to continuously improve their knowledge and skills to the benefit of quality patient care (Bigdeli, Adel-Mehraban & Namnabati 2019; Mamdouh & Samir 2022; Tyokwe & Naicker 2021). According to Madlabana-Luthuli (2019), inadequate performance assessment processes and postponed feedback might adversely affect nurses’ work lives. Fan and Zhu (2023) described performance assessment as an assessment strategy where evidence about learning is gathered through assigned performance tasks. Therefore, the organisation must ensure that the performance management process is appraised effectively and objectively to eliminate biases (Goel 2023). Hence, the study aimed to describe the ideal performance management process for professional nurses in the designated hospital in Gauteng province, South Africa.

Organisations should train managers to provide constructive criticism to their staff members (Hamdeen et al. 2022; Majidi et al. 2021). Nurse managers must have the same expertise as their subordinates to understand their challenges (Sepahvand et al. 2020). In the same vein, the findings of Al Aamri et al. (2024) showed that more than 60% of their respondents believed that the reason to conduct performance appraisal was only to evaluate the performance of employees without considering other factors such as job satisfaction, promotion and motivation. This necessitated further training for nurses to understand the process. A recommendation was made by Song et al. (2024) that organisations should establish a scientific performance appraisal system for nurses. Therefore, nurse managers should ensure that good communication exists and that timely feedback is delivered to nurses to strengthen their relationships. This study aimed to describe the ideal performance management process for professional nurses in the designated hospital.

### Problem statement

The performance management process should be a joint effort of the organisation and the nurses (Francis, Bin Ahmad & Binti Abdullah 2021). Not all nurses, unfortunately, understand what the assessment process entails (Katsinde, Tsododo & Katsinde 2021). If the process is poorly managed, nurses will be dissatisfied, and there will be high staff turnover. Challenges in the performance management systems are highlighted in the steps of the process, as it is indicated that managers often fail to observe their subordinates’ performance, provide delayed and unsupportive feedback, and do not appropriately address incentive systems for high achievers, while nurses also express dissatisfaction with the reward schemes linked to their performance appraisals (Francis et al. 2021; Homauni, Mosadeghrad & Jaafaripooyan 2021; Madlabana & Petersen 2020).

The researcher observed that in the designated hospital, professional nurses often complained about the steps in the performance interview, poor feedback afterwards and lack of incentives for their outcomes reached. It was thus unclear what the best experiences of professional nurses were on the performance process in the hospital.

### Paradigm

According to Polit and Beck (2022), assumptions derived from a worldview or paradigm are concepts scientists accept as the truth based on logic or reason. There is no need for scientific validation of the assumption. The constructivist paradigm’s assumptions were used in this research. The constructivist paradigm presupposes a subjectivist epistemology (knower and respondent co-create understandings), a relativist ontology (there are multiple realities) and a naturalistic (in the natural world) set of methodological procedures (Denzin & Lincoln 2003).

Concerns regarding the nature of reality are ontological assumptions (Klenke, Martin & Wallace 2015). The constructivist paradigm’s ontological premise highlighted the variety of perspectives on performance management. The researcher assumed that participants’ perceptions of performance management varied and that there were multiple realities (Denzin & Lincoln 2003).

The epistemological premise of the constructivist paradigm is that the participants and the researcher engage in an interactive process in which each influences the other (Mertens 2023). During focus group interviews, the participants and the researcher co-created the data.

The procedures of gathering and analysing data are determined by the methodological assumptions of research paradigms (Polit & Beck 2022). Data were collected using focus group discussions. The data analysis employed a thematic approach, forming themes and subthemes based on similar codes. The participants were free to explore and explain the ideal performance management process in a natural setting.

### Theoretical framework

**FIGURE 1:**
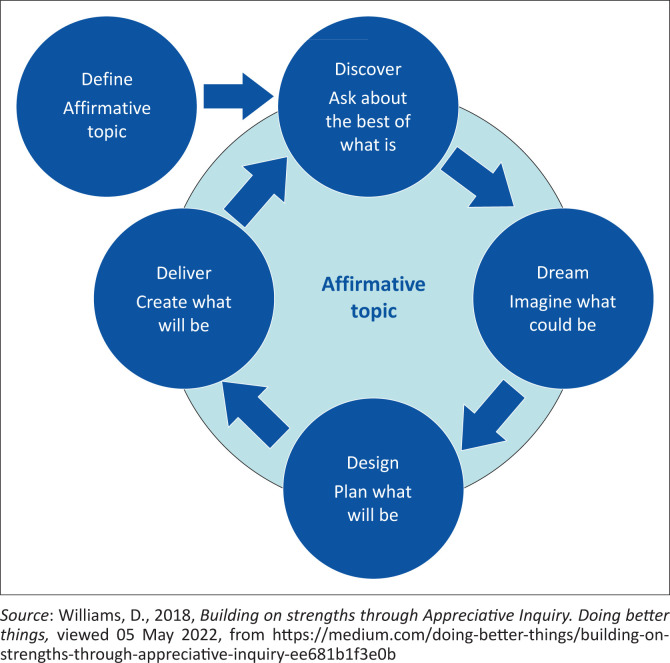
The 5-D appreciative inquiry model.

The 5-D Appreciative Inquiry (AI) model (refer to Figure 1), developed by Cooperrider and Srivasta (1987), was utilised as a theoretical framework to guide the data collection process and during the data analysis stage. LoBiondo-Wood and Haber (2018) describe a theoretical framework as a structure that creates a path for conducting the research. Merriel et al. (2022:1) describe AI as an encouraging change intervention organisations can use to enhance organisational culture. It encourages organisations to concentrate on the positive and investigate the best of ‘what is’ before considering ‘what could be’, opting for ‘what should be’, and experiencing ‘what can be’. It is an opportunity-based approach that enables managers and researchers to focus on what is right instead of identifying what is wrong in an institution. As Armstrong, Holmes and Henning (2020) indicated, AI is a legitimate way to deal with helping people and systems move from a deficit-based paradigm to a strength-based perspective.

## Research methods and design

### Study design

A descriptive, qualitative design using AI was adopted as the research design uses the principles of qualitative research as a positive framework to engage participants in transforming their environment (Reed 2007).

### The setting

The study was conducted in a private hospital in Gauteng, South Africa, with a bed capacity of 141 patients. The hospital renders medical, surgical, obstetrical, neonatal, paediatric, critical and emergency care services.

### Study population and selection of participants

At the time of the study, all the permanent professional nurses of the designated hospital formed part of the bi-annual, compulsory performance appraisal process. The study population comprised 56 professional nurses employed at the designated private hospital. Twenty-five professional nurses were selected purposively as they could provide rich data in line with the research questions (Grove & Gray 2019). They met the inclusion criteria requiring all potential participants to be professional nurses who were permanently employed at the hospital and had participated in at least one performance management cycle.

### Data collection

Focus group discussions were utilised in this study, as they allowed the participants to describe their experiences with one another. Participants interviewed in a focus group usually share similar roles and experiences (Holloway 2016). The researcher used focus group discussions to obtain the perspectives of a diverse group of professional nurses who participated in a performance management cycle at the designated hospital. The focus group discussion helped the participants clarify their views in a way that was less likely to occur in individual interviews (Grove & Gray 2019). It allowed professional nurses of different backgrounds to share their experiences. Focus group discussions took place during ‘off duty’ times and were arranged between the researcher and participants to ensure services rendered were not affected. Each focus group comprised five professional nurses, and the focus group discussions were conducted over 3 weeks. The focus group discussions were conducted in a private boardroom assigned to the researcher by the hospital management to ensure participant privacy. Participants were requested to keep the information shared during the focus group discussions confidential. Grove and Gray (2019) stated that confidentiality and comfort may result in richer dialogue and data. Each focus group discussion lasted between 60 min and 80 min.

The interview guide was drawn from the 5-D AI model concerning the performance management of professional nurses. The following questions guided the interview process; ‘What does performance management mean to you?’ (define). ‘What are the best parts of the current performance management process that you value?’ (discovery). ‘What are your wishes for the performance management process in the hospital?’ (dream). ‘What are the best steps to ensure the ideal performance management process?’ (design). ‘How can these steps ensure that your vision of the performance management process is sustainable?’ (destiny). The researcher used probing during the focus group discussions to ensure that rich data were obtained from the participants (Robinson 2023).

The researcher facilitated the focus group discussions with the assistance of a research assistant. The research assistant captured the information that could not be audio recorded in field notes, that is, nonverbal communication and seating of the participants. During the first meeting with the participants, the researcher described the study, explained AI and asked if they were interested in participating. They then completed the information and consent document. The researcher asked the questions, and the participants described their positive personal experiences with the current performance management process. The insights from the discovery phase helped the participants envision a desired future and co-create and share ideas about potential ways to bring what was envisioned into being. During the dream phase, all participants were asked to discuss their ideal dream for a performance management process. During the design and destiny phases, the participants were asked to discuss the actions to be implemented to meet their desired expectations regarding the performance management of professional nurses.

The focus group discussions were audio-recorded with the participant’s permission. Audio recordings enable the researchers to revisit the data collected during the transcription (Osborne & Grant-Smith 2021). The researcher continued discussions with participants over the 3 weeks of data collection until data saturation was reached (LoBiondo-Wood & Haber 2018).

### Data analysis

The 5-D model of AI guided the deductive data analysis process; however, all the other data that did not fit the AI model for analysis were analysed inductively. Five stages of data analysis were followed:

Step one – familiarisation: The audio recordings and field notes of the focus group discussions were transcribed. The transcripts were checked for accuracy by reading each focus group discussion transcript while listening to the audio recording. Anonymising of participants was ensured in that their names and places were not identifiable to maintain confidentiality (Gibbs & Flick 2018). Participants were then referred to as Participant 1, Focus Group Discussion 1, Participant 2 and Focus Group Discussion 2. Step two – identifying a thematic framework: Once the researcher was familiar with the data, themes were developed. Step three – coding: The researcher coded the transcripts using in-vivo codes according to the domains of the AI model (deductive analysis) and did thematic coding (inductive analysis) to analyse data that could not be analysed using the 5-D AI model. Stage four – mapping: The researcher assembled similar codes and created subcategories. Alignment of similar subcategories was then carried out to form categories. Stage five – interpretation: The researcher summarised the coded data, excerpts from the transcripts and information related to the research question and presented it as the study findings.

### Trustworthiness

The Lincoln and Guba (1985) framework, as described by Polit and Beck (2022), was utilised to ensure trustworthiness. The credibility of the findings was ensured through data saturation (Ahmed 2024). The research assistant took field notes to capture non-verbal cues and ensure that all pertinent data were analysed. To ensure confirmability, the researcher, through ongoing reflection, verified that the findings were the voice of the participants and not his own experiences influencing the participants. Dependability was ensured when data were analysed meticulously and consistently, and the methods used were clearly described. The researcher fully described the context of the study to qualify readers to weigh the applicability and transferability of the findings to other contexts.

### Ethical considerations

All study participants gave informed consent voluntarily. Ethical clearance was obtained from respective research ethics committees: the private hospital group (CRIP-10052023/15) and the University of Pretoria (71/2023). Permission to collect data from the participants was sought from the private hospital group and the management of the designated hospital. All procedures followed the ethical principles as informed by the Belmont report (LoBiondo-Wood & Haber 2018). The researcher ensured that the participants’ rights to freedom from harm and discomfort were respected. They were not subjected to harm, and their right to protection from exploitation was ensured. The information provided was not used against them, and their human dignity was respected. The participants were allowed to withdraw at any time without negative consequences. Their right to full disclosure was protected, and the research process was discussed thoroughly to enable them to make informed decisions. The researcher ensured that the participants were treated fairly and that their privacy rights were respected. Confidentiality was ensured by using pseudonyms. Data were stored safely on a password-protected computer. No data were shared in reports that may have led to them being associated with the data.

## Results

### Demographic profile of participants

There were 25 professional nurses in total. Twenty-one had a diploma in nursing, and four had a 4-year bachelor’s degree in different fields of nursing. Five participants had between 1 and 10 years of experience, and 20 had 11 or more years of experience.

### Themes

Five themes and 13 subcategories emerged from data analysis.

### Theme 1: Knowledge of the performance management process

The theme relates to the ‘define phase’ of the 5-D AI Model. The three sub-categories under this theme include setting goals and rating performance, evaluating the achievement of goals and enabling professional nurses’ development. These subcategories addressed how participants understood and described the definition of performance management.

#### Setting goals and rating performance

According to participants, the managers set goals and explain targets that employees are expected to meet. Performance ratings are, therefore, determined based on the agreement between the manager and the employee. The following quotations support this:

‘Performance management is that period when nurses meet with their managers and discuss expected goals. They usually explain what needs to be achieved and then rate us based on what we have produced.’ (P8, Female PN, FGD2)

‘Okay, so performance management, what I understand by that is how well we reach our goals in the ward or in a workplace, like let’s say our goal is to ensure that nursing care is done correctly, like our patients are happy, and we will start rating ourselves.’ (P24, Female PN, FGD6)

#### Evaluating the achievement of goals

Participants described the definition as an evaluation process to determine if set goals were achieved and as a means of measuring employee competence. The participants verbalised the following:

‘It’s when we are getting evaluated and rated every six months, I think … we normally get rated, and if we performed well, we then get good increments, and our salaries improve.’ (P3, Female PN, FGD3)

‘Okay, for what I understand, performance management is used to check our competencies in our daily practice. We get evaluated and rated based on what we produce.’ (P4, Female PN, FGD6)

#### Enabling professional nurses’ development

A participant described performance management as a process that allows the manager and the professional nurse to identify gaps. When gaps are identified, opportunities for training arise to ensure that the employee improves. Participants saw it as an opportunity for the success and growth of employees:

‘If you see that there is a lack of skills, then you can sort of like make sure that the person goes for training or continuous training.’ (P25, Female PN, FGD6)

‘It also provides an opportunity for study opportunities for those that want to grow in the field of nursing.’ (P5, Female PN, FGD4)

### Theme 2: Factors which might promote positive participation in the performance management process

This theme relates to the ‘discovery phase’ of the 5-D AI model and addresses the aspects of performance management that the participants appreciated. These aspects refer to the positive influence of unit managers, the incentives the participants received, how the managers treated them and how developing skills, training and leadership positions motivated the participants to perform well.

#### Positive attributes and employee support in the performance management process by the managers

Managers play an essential role in influencing and motivating employees. Participants regarded managers’ support as crucial and said that their role in initiating positive change may have contributed to the success of the ideal performance management process, as indicated by the following quotations:

‘Yes, so what they do, they will see where you do well then, they will put you there to manage that program, and all your trainings will be in that direction to empower you there. So, even though it doesn’t translate into cash, they give you extra empowerment.’ (P6, Female PN, FGD3)

‘So, I was happy because that unit manager didn’t look at how or what. She was rating us according to the way we were working. I was happy because she gave me the rate I was deserving to get.’ (P18, Female PN, FGD5)

#### Incentives as a motivational strategy for good performance

Motivation is always a critical factor in producing good results. Participants appreciated the incentives that motivated them to perform well. Through their life experiences, they mentioned what aspects played a significant role in ensuring that they stay motivated:

‘So, I went to do my theatre training, and we were eleven in class and out of eleven, I was the only one that passed that course. Yes, they paid for everything, they even increased the salary when I came back with the certificate, yes! They acknowledged everything.’ (P2, Female PN, FGD2)

‘I was also recognised by that unit manager. She even sent my recognition to the college, and then I got cum laude, and I was proud of myself. I passed, and I was already at the college as a student then and was sent by the company due to my exceptional performance.’ (P2, Female PN, FGD6)

### Theme 3: Changes in perspectives that might contribute to an ideal state of the performance management process

This theme focusses on the participants’ dreams of what ideal performance management should look like. The participants focussed mainly on what can make the process run seamlessly. Most participants reported a fair and equitable performance management process. However, a few incidents relating to a negative work environment, mostly criticism, were noted as barriers to effective communication. They mentioned the need for mutual respect between the managers and professional nurses and a fair performance management process to prevent bias and favouritism. Participants voiced the need for the managers to lead by example and be transparent in assessing and rewarding performance.

#### Envisioned process of performance management:

Most participants stated the importance of having a fair and transparent process to ensure productivity. Threatening environments that promoted negative criticism were seen as a barrier to effective communication. Aspects of mutual respect between managers and employees were raised. They further stated that the avoidance of bias and favouritism will ensure that a fair process exists. Evaluation of employees must be based only on performance rather than other personal factors:

‘I think my wish is that there must be a two-way communication; it must be interesting like it must be not a situation wherein you feel like I’m sitting with my manager, and you are afraid to talk. It must be a situation where you are free to talk to express yourself, and there must be less criticism. It must be educative like you are free to express yourself and you enjoy instead of coming there like maybe crying feeling like you are not doing anything.’ (P21, Female PN, FGD6)

‘My dream will be to be rated correctly for the job performance we produce. You know we work very hard, but you find that we all get to be allocated the same rate … why so? I know that I put so much effort and work, but you get to be rated like everyone else, and that’s wrong. So, my dream will be that we get rated correctly and also awarded for that performance.’ (P2, Female PN, FGD3)

#### Envisioned nurse managers’ positive behaviours:

Managers’ positive attitudes contributed to the team’s success in the unit. This, in turn, promotes the high performance of employees when they learn from their leaders. Participants verbalised the need for the managers to lead by example and be transparent:

‘Transparency! You know, if you are going to be in leadership … you need to lead by example… Show me that you know how to do it, be hands-on! You know it’s so much easier to do something that you saw than something you were just told. Don’t give us a tool or a SOP or guideline. Just be present and say this is how we’d do it here. [*laughin*]).’ (P7, Female PN, FGD3)

‘[… *I*]f you come in and you don’t even recognise us, and we have a problem, but we can’t even talk to you … you know, sit down, and then you just wanted to say, “My rule my ways”.’ (P6, Female PN, FGD3)

### Theme 4: Goal-directed strategies for enabling a positive performance management process

This theme relates to the ‘design phase’ of the 5-D AI Model. It focusses on interventions that should be implemented to ensure that the process becomes feasible. It comprises the following subcategories: training for performance management, cooperative development of tools for performance management and implementing positive change.

#### Training for performance management

Training emerged as an essential aspect for most participants. It was suggested that nurse managers undergo training in performance management to ensure that the process remains fair for all employees. Other participants mentioned that managers and employees should have the same training provided to ensure that they all understand the correct procedure:

‘The manager needs to know exactly what they are looking for, and they need to understand themselves … They need to undergo training for the JPM [*Joint Performance management*] and how to rate so they can easily explain it to the professional nurses, the person they are rating.’ (P19, Female PN, FGD5)

‘But maybe with time they will have a change of mindset so I will say training may be beneficial for all the managers.’ (P5, Female PN, FGD6)

#### Cooperative development of performance management tools

The importance of cooperative development of performance development tools by both employees and their managers was raised by most participants. Documents need to be unit-specific and not fixed. Participants also reported that the performance management tool needs to be individualised based on the nurse’s experience. Furthermore, changes in performance management policies are required:

‘I think currently now, we are using a standardised document which is standard across all the departments. I think, obviously, our dreams being different. A specialised unit needs to have its own like job management thing separate because it’s specialised. The goals there must actually relate to what the unit does. For example, even if we mention the skills, it must be relevant to what the unit is, which will be different, for example, the one in maternity and to the one in casualty.’ (P7, Female PN, FGD3)

‘If you break it down correctly and make it understandable, put it in a language that an HR person won’t just understand but also us as clinical as we are, then at least you can get participation from the person who is supposed to perform because now you know exactly what is.’ (P11, Female PN, FGD4)

‘They must be very specific with the questions they put on the tool, yes. My dream was to review the policy.’ (P4, Male PN, FGD6)

#### Implementing positive change

For every organisation to achieve exceptional results, there should be mutual collaboration between the managers and the employees in implementing positive change. Good relationships between managers and employees will strengthen the performance management process as there will be mutual trust. Transparency in the performance management process will ensure that every employee is familiar with the set objectives and goals. Avoidance of biases by the managers will motivate employees to perform well:

‘If unit managers stop favouritism, everything is going to be okay, yeah, they must just treat us the same.’ (P17, Female PN, FGD5)

### Theme 5: Measures to preserve an ideal state of the performance management process

This theme addresses the ‘deliver phase’ of the 5-D AI Model and, therefore, focusses on positive solutions to ensure that the performance management process remains sustainable. Performance management should be the responsibility of all stakeholders of different disciplines in the designated hospital. The three subcategories that emerged from the data include stakeholder involvement, effective communication and enabling relationships.

#### Stakeholder involvement

Top management, together with unit managers, needs to manage and monitor the process of performance management. They must seek to understand what staff concerns are. Hospital management and head office should be involved in the process so that staff challenges can be addressed and attended to:

‘Line managers must be there, and also the top [*management*] those [*who*] are making things to happen. There must be there. So that they change things and understand what the people at the ground complain about then make it easier for them to get those policies reviewed.’ (P9, Female PN, FGD3)

‘Unit managers to be monitored by matron that they are rating based on our key performance areas.’ (P16, Female PN, FGD4)

‘The HR they must be involved because they are the ones whose dealing with the money. They must give us the increase based on our performance. They must not hear somebody said “Marietjie, you can give her two, and then they give me 2” Nooo! [*Nodding her head and laughing so loud*].’ (P3, Female PN, FGD3)

#### Effective communication

Policymakers must ensure that the document on performance management is user-friendly and easy to understand. Furthermore, there must be ongoing communication between the hospital management and the head office to address challenges:

‘I’m trying to say policy regarding performance management must be unit specific because I’ve seen it being generalised, and we are being misunderstood where you need to explain your points. Yes!’ (P3, Male PN, FGD3)

‘So, the management is the one that needs to hold this thing together and to follow it up because it’s their tool. They see what’s goes wrong, they see what lacks. So, they are the one to convey it to the Head office and also to follow it up.’ (P16, Female PN, FGD4)

#### Enabling relationships

Participants indicated that all the managers within the hospital group should form an organisation where they can share ideas that will help improve the system and maintain the improvement of the performance management process. They also felt strongly that doctors should be included in the performance management process and that units should select one nurse to represent them in meetings about the performance management process:

‘Managers need to form an organisation of some sort to write and say as the hospital group. We need to change this policy because it must go together with whatever that they are doing according to their departments.’ (P5, Female PN, FGD2)

‘Okay, I think even the doctors are to be involved because we work with them most of the time, one-on-one.’ (P20, Female PN, FGD5)

## Discussion

Participants described the performance management process as the meeting between managers and professional nurses to discuss expectations, achievements and ways to address poor performance. During such meetings, clear instructions and feasible expectations must be stated (Sides & Cuevas 2020). The meetings could be used to encourage professional nurses to meet personal goals and the organisation’s performance goals (Nduati & Wanyoike 2022).

Participants viewed performance management as a series of processes to implement the achievement reward system of the designated hospital. Ayi and Korang (2024) described a reward system as a strategic approach to ensure employees remain motivated in doing their jobs. This statement is supported by Lazarova, Thomas and Ferndale (2021), who emphasised that performance management denotes employees’ compensation and future job directions. Participants agreed that performance management strength is when gaps are identified, and further training is provided as a corrective measure. This view is supported by the description of Atmaja, Zaroni and Yusuf (2023) that the process should assist employees and managers in identifying competency gaps that should be addressed in the following performance management cycle. Nguyen and Nguyen (2023) maintained that managers need to plan diversified and effective training to assist employees in developing their capacity to assist with their career paths and succession planning.

The participants desired influential managers who could enable them to enjoy satisfying work-life experiences. Nguyen and Nguyen (2023) suggested that managers should create a corporate culture that promotes positive work-life and good relationships between managers and subordinates. This view is that managers need to support and celebrate their teams while appreciating their accomplishments because, when employees are motivated, they tend to approach delegated tasks with great ease and enthusiasm (Roussel, Harris & Thomas 2020). Employee empowerment by managers was another peak experience that emerged from the discussions. Empowered employees positively influence organisational performance (Hakim & Supriyatno 2023). Muller and Bester (2016) agree that a leader should not only lead the way for their followers but also provide support throughout the process. Hakim and Supriyatno (2023) assert that empowering employees is achieved when leaders involve them and provide quality guidance.

The participants appreciated being nominated and awarded for good performance. Rewards can foster creativity and motivation among employees (Mdhlalose 2024). Lui and Lui (2022) suggested that meaningful incentives should be beneficial in healthcare as this can encourage employees to perform well and improve client satisfaction. Sugiarti (2023) states a strong link exists between motivation to improve the practice and the willingness to participate in further training.

Participants described an equitable performance management process as being free from managers’ biases. Favouritism is associated with employees’ negative experiences of performance management (Pearce & Wang 2023). The participants also recommended that the managers of professional nurses should at least possess similar professional qualifications as their subordinates and attain the required knowledge and skills, ensuring that they have the qualifications and competence to perform their performance roles. The findings of Ofei, Paarima and Kwashie (2020) correspond with the recommendation that managers should be equipped with appropriate skills and knowledge to perform their performance management roles. Unfortunately, the study findings showed that some managers lacked the knowledge and skills to perform the performance appraisals of their professional nurse subordinates. Wärnich et al. (2018) stressed the need for managers to be well-trained in performance management to conduct fair appraisals. The recommendations by Qawasmeh et al. (2024) were proposed to enhance the effectiveness of human resources management, including investing in managerial training programmes to implement transparent performance appraisals. Participants suggested that stakeholders in performance management, such as nurse managers, the human resources department and even doctors and patients, are expected to work together to ensure that challenges associated with performance management are appropriately addressed (Wärnich et al. 2018). In support of this statement, White, Aiken and McHugh (2019) stated that developing a performance management system that permits a root-cause analysis may ensure that challenges are managed to benefit all involved.

Participants reported that the need for policy review emanated from the pitfalls of the current performance management process. Musodza, Cishe and Mapangwana (2021) suggest that policymakers consult users before they review and implement policies. Noordiatmoko and Riyadi (2023) indicate when policies are not consultative, their implementation may be unsuccessful. Policy-decision makers can improve clear guidelines and procedures regarding performance appraisal and approaches that progress job satisfaction among nurses (Al Aamri et al. 2024). The participants recommended that there must be constant communication among stakeholders in performance management. Stacho et al. (2019) concluded that open communication among stakeholders may enhance employee engagement in performance management and increase the organisation’s competitive edge. When employees and managers are engaged in open communication, they can quickly improve and share organisational goals (Kalogiannidis 2020).

### Recommendations

Recommendations are based on the findings of the study. The following recommendations are for the practice: Management should recognise professional nurses’ role in quality patient care and ensure a sound reward system by providing regular feedback, fostering a supportive environment, and practising fair and transparent decision-making. Policies should be reviewed to ensure the appraisal tool is unit-specific, fair and easy to understand. The performance management process is to be introduced early. Hospital management should establish clear and achievable performance standards, implement a comprehensive evaluation system, promote a culture of continuous improvement and provide constructive feedback and support.

The following areas for future research are proposed: To explore the challenges surrounding the performance management needs of nurse managers and the benefits of engaging other relevant stakeholders in performance management. In addition, regulatory guidelines or an effective model should be developed to guide performance management and ensure sustainability.

### Study limitations

The study had limitations as it was limited to professional nurses of one hospital in Gauteng province in South Africa.

## Conclusion

The findings of this study revealed a major need for proper training in performance management for both managers and professional nurses, organisations to have a sound reward system in place to recognise high achievers and all stakeholders to work together to ensure that the process remains customised and effective. It is rational to deduce that support from managers and top administration assists professional nurses in meeting their development goals. Fairness, transparency and visibility of all relevant stakeholders during the performance management process will guarantee that challenges are addressed. This will ensure that professional nurses remain motivated to deliver quality patient care.
